# Molecular mechanisms of thermal resistance of the insect trypanosomatid *Crithidia thermophila*

**DOI:** 10.1371/journal.pone.0174165

**Published:** 2017-03-22

**Authors:** Aygul Ishemgulova, Anzhelika Butenko, Lucie Kortišová, Carolina Boucinha, Anastasiia Grybchuk-Ieremenko, Karina A. Morelli, Martina Tesařová, Natalya Kraeva, Danyil Grybchuk, Tomáš Pánek, Pavel Flegontov, Julius Lukeš, Jan Votýpka, Márcio Galvão Pavan, Fred R. Opperdoes, Viktoria Spodareva, Claudia M. d'Avila-Levy, Alexei Yu. Kostygov, Vyacheslav Yurchenko

**Affiliations:** 1 Life Science Research Centre, Faculty of Science, University of Ostrava, Ostrava, Czech Republic; 2 Biology Centre, Institute of Parasitology, Czech Academy of Sciences, České Budějovice (Budweis), Czech Republic; 3 Coleção de Protozoários, Laboratório de Estudos Integrados em Protozoologia, Instituto Oswaldo Cruz, Fundação Oswaldo Cruz, Rio de Janeiro, Brazil; 4 Instituto de Biologia Roberto Alcântara Gomes, Departamento de Ecologia, Universidade Estadual do Rio de Janeiro, Rio de Janeiro, Brazil; 5 Institute for Information Transmission Problems, Russian Academy of Sciences, Moscow, Russia; 6 Faculty of Sciences, University of South Bohemia, České Budějovice (Budweis), Czech Republic; 7 Canadian Institute for Advanced Research, Toronto, Ontario, Canada; 8 Department of Parasitology, Faculty of Science, Charles University, Prague, Czech Republic; 9 Laboratório de Mosquitos Transmissores de Hematozoários, Instituto Oswaldo Cruz, Fundação Oswaldo Cruz, Rio de Janeiro, Brazil; 10 de Duve Institute, Université Catholique de Louvain, Brussels, Belgium; 11 Zoological Institute of the Russian Academy of Sciences, St. Petersburg, Russia; 12 Department of Pathology, Albert Einstein College of Medicine, Bronx, New York, United States of America; 13 Institute of Environmental Technologies, Faculty of Science, University of Ostrava, Ostrava, Czech Republic; Sichuan University, CHINA

## Abstract

In the present work, we investigated molecular mechanisms governing thermal resistance of a monoxenous trypanosomatid *Crithidia luciliae thermophila*, which we reclassified as a separate species *C*. *thermophila*. We analyzed morphology, growth kinetics, and transcriptomic profiles of flagellates cultivated at low (23°C) and elevated (34°C) temperature. When maintained at high temperature, they grew significantly faster, became shorter, with genes involved in sugar metabolism and mitochondrial stress protection significantly upregulated. Comparison with another thermoresistant monoxenous trypanosomatid, *Leptomonas seymouri*, revealed dramatic differences in transcription profiles of the two species with only few genes showing the same expression pattern. This disparity illustrates differences in the biology of these two parasites and distinct mechanisms of their thermotolerance, a prerequisite for living in warm-blooded vertebrates.

## Introduction

The order Trypanosomatida unites obligatory parasites with a single flagellum and a single kinetoplast, a structure containing mitochondrial DNA in the form of concatenated minicircles and maxicircles [1–3]. This order is further sub-divided into two groups: the monoxenous (= one host) parasites of insects and the dixenous (= two hosts) species alternating between an insect vector and a vertebrate or a plant host during their life cycle. The latter group is particularly important as it encompasses *Leishmania* and *Trypanosoma*, pathogens responsible for various diseases currently affecting over 22 million people worldwide [4–6]. While dixenous trypanosomatids were extensively studied, their monoxenous kins remained largely neglected and little was known about their biodiversity, biochemistry, cellular biology, and genetics [4,7,8]. Nevertheless, they are crucial for tracking the evolution of parasitism [9], have significant impact on their hosts' "physiological fitness" [10], and may affect insects' communities in a global way [11,12]. Moreover, monoxenous trypanosomatids have been reported as co-infecting agents with *Leishmania* spp. in immunocompromised and even in immunocompetent patients [13–15].

Most of the formally recognized monoxenous species were described based on morphology, life cycle, and host specificity [1,16,17]. However, it became evident that even combined these criteria cannot provide sufficient phylogenetic resolution and that molecular and biochemical data are needed for accurate taxonomy [18–20]. Thus, molecular analyses have become widely used for the purpose of classification and re-classification of trypanosomatids [8,21–23]. One of the prominent examples concerns endosymbiont-containing trypanosomatids originally classified as *Crithidia*, *Blastocrithidia*, and *Herpetomonas* spp. [24–26]. This group was found to be monophyletic [27] and all these species were relocated into two new genera: *Angomonas* and *Strigomonas* [19]. Along with a recently described genus *Kentomonas*, these genera are now united into a new subfamily, Strigomonadinae [23]. The genus *Wallacemonas* is another illustrative example. It is composed of species that were previously classified as *Leptomonas* and *Wallaceina* (synonymized with *Crithidia*) but revealed to be phylogenetically related and sharing common molecular traits [28–30].

The genus *Crithidia* with its type species *C*. *fasciculata* accommodates monoxenous parasites of insects represented by choanomastigotes. The original illustrations of Léger also depicted epimastigotes representing another component of mixed infection which was subsequently classified as *Blastocrithidia* [7,31,32]. Subsequent phylogenetic analyses revealed that many *Crithidia* spp. do not cluster with the type species and, in fact, belong to different genera of Trypanosomatidae [28,33,34].

Several representatives of the genera *Crithidia* and *Herpetomonas* can withstand elevated temperature [35–37]. This can be viewed as a pre-adaptation to the dixenous life cycle–a trypanosomatid flagellate must be able to survive in the aggressive environment of the warm-blooded host [13]. One of such trypanosomatids, *Crithidia luciliae thermophila* Roitman et al., 1977 was isolated from a reduviid bug *Zelus leucogrammus* in Brazil [38]. It was proposed as a subspecies of *C*. *luciliae* (Strickland, 1911) Wallace et Clark, 1959 following the recommendation of F. G. Wallace not to describe trypanosomatids having only biochemical/physiological differences as separate species [16,39]. These two sub-species can be distinguished biochemically (utilization of sorbitol, mannitol, ribose, galactose, and cellobiose) or by temperature resistance as *C*. *luciliae* cannot grow at elevated temperature [38].

In the present work, we demonstrate that *Crithidia luciliae thermophila* is not a subspecies, but a separate species *C*. *thermophila*. We also investigated molecular mechanisms governing thermal resistance of this species. For that purpose, we compared transcriptomic profiles of flagellates cultivated at low and elevated temperature. Transcription of genes involved in sugar metabolism and mitochondrial stress protection was significantly upregulated at high temperature.

## Materials and methods

### Trypanosomatids strains and cultivation

All eight strains used in this study (Table 1) are deposited at Fiocruz Protozoa Culture Collection (COLPROT), Rio de Janeiro, Brazil, and can be requested at http://colprot.fiocruz.br [40]. The initial taxonomic identification was provided by the original depositors. Trypanosomatids were grown at 23°C in liquid Brain Heart Infusion (BHI) medium (Sigma-Aldrich, St. Louis, USA) supplemented as described previously [41] and passaged weekly. For growth curves at different temperatures (23°C and 34°C), flagellates were seeded at a concentration of 30,000 cells per ml, and counted in triplicates at days 1, 3, and 5.

**Table 1:** Species, strains and isolates analyzed in this study.

COLPROT	Alt. ID	Original name	Reclassification	Host	Year	Locality
**018**	ATCC 30818	*Crithidia hutneri*	*C*. *thermophila*	*Cosmoclopius* sp. (Hemiptera)	1975	Mambai GO, Brazil
**053**	ATCC 14765, 0258	*C*. *luciliae*	*C*. *fasciculata*	*Phaenicia sericata* (Diptera)	1958	Minneapolis MN, USA
**054**	ATCC 30817	*C*. *luciliae thermophila*	*C*. *thermophila*	*Zelus leucogrammus* (Hemiptera)	1973	Goiânia GO, Brazil
**056**	NA	*Crithidia* sp.	*C*. *thermophila*	*Zelus leucogrammus* (Hemiptera)	1991	Belo Horizonte MG, Brazil
**676**	ATCC PRA-346	*C*. *confusa*	*C*. *thermophila*	*Largus* cf. *cinctus* (Hemiptera)	2009	Alajuela Province, Costa Rica
**688**	ATCC 30818	*C*. *hutneri*	*C*. *thermophila*	*Cosmoclopius* sp. (Hemiptera)	1975	Mambai GO, Brazil
**689**	ATCC 30817	*C*. *luciliae thermophila*	*C*. *thermophila*	*Zelus leucogrammus* (Hemiptera)	1973	Goiânia GO, Brazil
**703**	NA	Trypanosomatidae sp.	*C*. *thermophila*	*Cyrtoneuropsis conspersa* (Diptera)	2015	Rio de Janeiro RJ, Brazil

### Light and electron microscopy

Light microscopy of Giemsa and DAPI (4',6'-diamidino-2-phenylindole; Sigma-Aldrich) stained smears was done as described elsewhere [42]. Standard measurements were performed on Giemsa-stained smears for 50 cells in each biological replicate, and expressed in μm. Methods used for scanning electron microscopy (SEM) and high-pressure freezing transmission electron microscopy (HPF-TEM) were described elsewhere [30]. HPF-TEM images were captured using Orius SC1000 CCD camera (Gatan, München, Germany).

### DNA extraction, PCR amplification, and sequencing

Total DNA was extracted from cultured trypanosomatids at mid-log growing phase (2 x 10^7^ cells per ml) using the Wizard Genomic DNA Purification kit (Promega, Madison, USA) or DNeasy Blood and Tissue kit (Qiagen GmbH, Hilden, Germany) according the manufacturers' protocols. The 18S ribosomal RNA, glycosomal glyceraldehyde 3-phosphate dehydrogenase (gGAPDH), and spliced leader (SL) RNA genes were amplified as described previously [43–45]. 18S rRNA and gGAPDH PCR products were sequenced directly. The SL amplicons were cloned using the InsTA PCR Cloning kit (ThermoFisher Scientific, Waltham, USA). The sequences were deposited under the following GenBank accession numbers: KY264921 –KY264929 (SL RNA), KY264930 –KY264936 (gGAPDH), and KY264937, KY364901 (18S rRNA)

### Phylogenetic analyses

The previously built alignments of 18S rRNA and gGAPDH genes [28] were supplemented by several sequences including those of the strains under study. Then ambiguously aligned positions in 18S rRNA gene alignment were removed manually in BioEdit [46] and the alignments of the two genes were concatenated. Maximum likelihood and Bayesian trees were reconstructed in Treefinder v. 03.2011 [47] and MrBayes 3.2.6 [48] as described before [28].

### Whole transcriptome sequencing, assembly, and annotation

The axenic culture of the isolate COLPROT 689 was cultivated at 23°C or 34°C for 84 h. Total RNA was isolated from 5 × 10^7^ cells using the RNeasy Mini kit (Qiagen GmbH) according to the manufacturer’s instruction. The cDNA libraries were sequenced for three independent biological replicates with 100 nt paired-end reads on the Illumina HiSeq 2000 platform (Macrogen Inc., Seoul, Republic of Korea). Prior to assembly, RNA-seq reads were subjected to adapter and quality trimming using Trimmomatic v. 0.32 [49] with following parameters: illuminaclip: TruSeq3-PE-2.fa:2:20:10:8:true; leading: 3; trailing: 3; slidingwindow: 4:15; minlen: 75. All other parameters were left as default. The Trinity assembler v. 2.0.6 [50] was used to reconstruct the transcriptome *de novo* with the following settings: min_kmer_cov = 1, min_contig_length = 200. The resulting assembly of 250 million reads had the average contig length of 1,477 bp. Over 95% of reads were mapped to the assembled contigs.

### Differential gene expression analysis

Differential gene expression analysis was performed using the RNA-Seq tool in CLC Genomics Workbench 9.0.1 (Qiagen GmbH). Trimmed reads were mapped to the assembled transcriptome with the following parameters: maximum number of mismatches = 2; minimum fraction of read length mapped = 0.9; minimum identity within the mapped sequence = 0.95; maximum number of best-scoring hits for a read = 30. The expression values for each transcript were calculated as Reads Per Kilobase of transcript per Million mapped reads (RPKM). To identify transcript sets that are differentially expressed at low and elevated temperature, the 'Exact Test' for two-group comparisons [51] implemented in the Empirical analysis of DGE tool was applied. Transcripts with expression fold change over 1.5 and an FDR-corrected p-value below 0.05 were chosen for further analyses. Differentially expressed transcripts (N = 108) were annotated using BLAST with E value cutoff of 10^−7^.

Coding regions within transcripts were predicted using TransDecoder v.3.0.0 (http://transdecoder.github.io) with default settings. In order to find common genes differentially expressed at elevated temperature, protein sequences corresponding to the predicted coding regions were used as an input for OrthoFinder v.0.7.1 [52] along with the annotated proteins of *Leishmania major*, *Leptomonas seymouri*, and *Crithidia fasciculata* downloaded from the TriTrypDB v.9.0 database [53].

## Results and discussion

### Morphological and ultrastructural characterization

**Fig 1:**
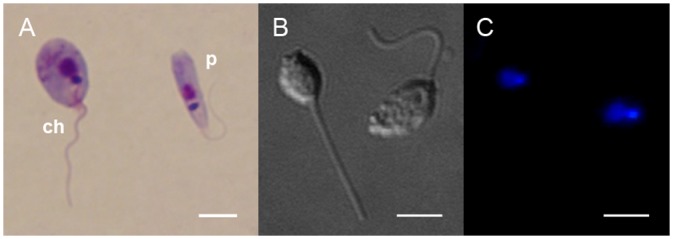
Light microscopy of the isolate COLPROT 054. **(A)** Giemsa-stained pro- and choanomastigotes are shown. Differential interference contrast **(B)** and fluorescent **(C)** microscopy of the DAPI-stained slides demonstrate presence of the nucleus and kinetoplast. Scale bars are 2.5 μm.

Inspection of the axenic cultures COLPROT 054, 056, 689, and 703 by light and electron microscopy did not uncover any species-specific traits (Fig 1 for isolate COLPROT 054). The morphology appeared to be the same as for the previously described *C*. *confusa* [20].

Cells cultivated at different temperatures displayed some morphological changes (see below). *C*. *thermophila* grown in BHI at 23°C ranged from typical promastigotes to choanomastigotes (Fig 1A, labeled *p* and *ch*, respectively). The length and width of these cells varied between 3.4 and 6.6 μm (5.2 ± 0.7 μm, hereafter N = 150), and 0.9 and 3.0 μm (1.7 ± 0.4 μm), respectively. The distance from the nucleus to the anterior end of the cell measured from 1.2 to 3.1 μm (1.9 ± 0.3 μm), whereas the distance from the kinetoplast to the anterior end varied between 0.5 and 2.2 μm (1.4 ± 0.3 μm). The flagellum was always present and its length was between 0.9 and 7.6 μm (5.3 ± 1.3 μm). The kinetoplast disk was of typical shape and size, with the width between 420 and 964 nm (704 ± 141 nm, N = 32) and the thickness from 131 to 228 nm (175 ± 22 nm, N = 32).

**Fig 2:**
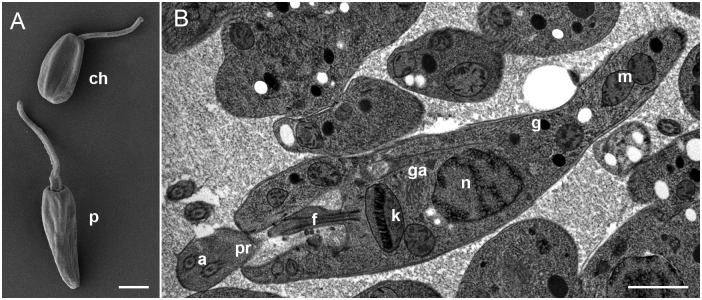
Electron microscopy of the isolate COLPROT 054. **(A)** Scanning electron microscopy, **(B)** high-pressure freezing transmission electron microscopy. The longitudinal sections reveal typical features of trypanosomatids such as axoneme (*a*), flagellum (*f*), glycosomes (*g*), Golgi apparatus (*ga*), kinetoplast (*k*), mitochondrion (*m*), nucleus (*n*), and paraflagellar rod (*pr*). Scale bars are 1 μm.

SEM revealed the same morphotypes–pro- and choanomastigotes (Fig 2A). TEM showed features of a typical trypanosomatid cell, e.g. an oval nucleus, a kinetoplast, a single Golgi apparatus, few glycosomes, a peripherally located reticulated mitochondrion with numerous cristae, and a flagellum with paraflagellar rod (Fig 2B).

### Comparison of *Crithidia* strains using standard molecular markers

When we started to characterize *C*. *luciliae thermophila* (COLPROT 054) in molecular terms, we found out that it was indistinguishable from *C*. *hutneri* (COLPROT 018) by 18S rRNA and gGAPDH gene sequences. The SL RNA gene sequences of these strains showed 5% difference, which is below the generally accepted interspecific threshold [44]. We ordered new replicas of the original strains of both species–ATCC 30817 (COLPROT 689) and ATCC 30818 (COLPROT 688)–and repeated the analysis which actually showed the same results. Afterwards, we searched the COLPROT collection and identified two additional strains (COLPROT 056 and 703), which proved to be same species as judged by sequences of all three genes. GenBank searches revealed that *C*. *confusa* ATCC PRA-346 also belongs to this species as judged by comparisons of the sequences of COLPROT 056, COLPROT 703, and ATCC PRA-346.

The situation with the nominal subspecies *Crithidia luciliae luciliae* (traditionally named just as *C*. *luciliae*) is no less confusing. The original culture deposited to ATCC under accession number 14765 was discontinued and substituted with another culture (ATCC 30258). We analyzed a replica of the ATCC 30258 and showed that it is different from *C*. *luciliae thermophila* but identical to *C*. *fasciculata* (strain Cf-C1) as judged by its 18S rRNA gene sequence. These results are consistent with previously published data on riboprinting profiles of *Crithidia* spp. (Table 1 in [54]). By this method, *C*. *lucilae* and *C*. *fasciculata* were indistinguishable, and so were *C*. *luciliae thermophila*, *C*. *hutneri*, and *C*. *confusa* (labeled then as aposymbiotic *C*. *deanei*). The similarity between *C*. *lucilae* and *C*. *fasciculata* was also reported upon redescription of the former species in 1959 [55]. The original description by Strickland [56] was based on a mixed infection presumably with *Herpetomonas* and *Blastocrithidia*, and thus could not be used for proper identification.

In sum, our results strongly support the following taxonomic revisions: First, C. *luciliae thermophila* is a biological species separate from and unrelated to *C*. *luciliae*, and the taxon must be raised in status and henceforth named *C*. *thermophila*. Further, *C*. *confusa* and *C*. *thermophila* are the same species, and *C*. *confusa* should be considered a junior synonym of *C*. *thermophila*, and *Crithidia luciliae* is a junior synonym of *C*. *fasciculata*. Finally, *C*. *hutneri*, as judged by its original description, differs from *C*. *thermophila* in metabolic properties. The fact that the culture of *C*. *hutneri* preserved in ATCC and COLPROT represented *C*. *thermophila* was likely due to a laboratory error before submission to ATCC.

### Phylogenetic analysis

**Fig 3:**
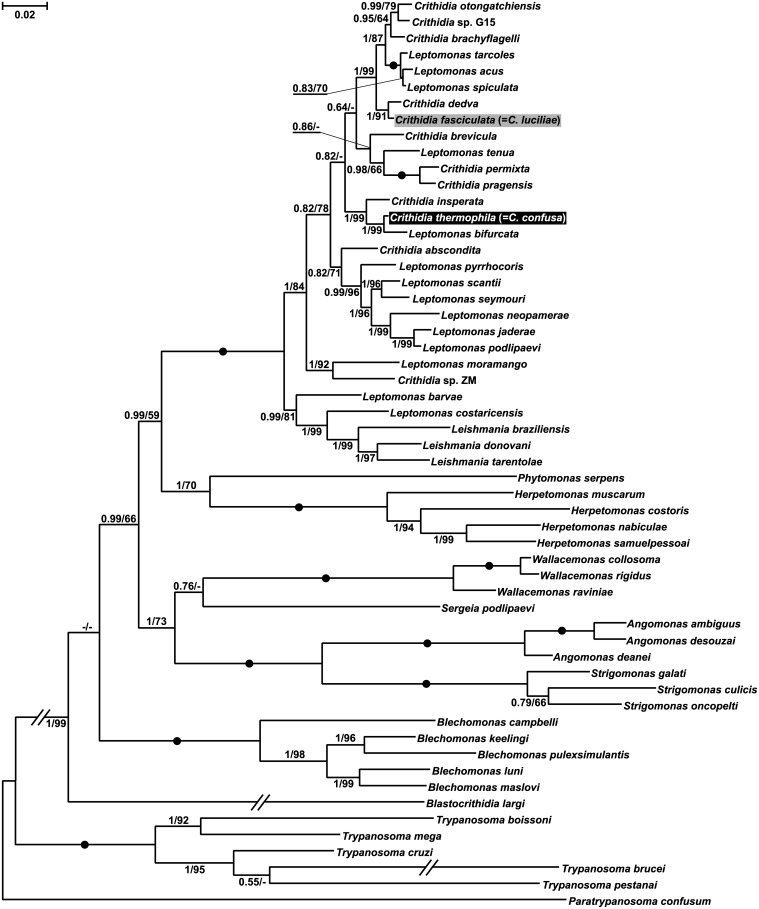
Maximum likelihood phylogenetic tree of Trypanosomatidae. This tree is based on concatenated 18S (SSU) rRNA and glycosomal glyceraldehyde-3-phosphate dehydrogenase (gGAPDH) gene sequences and inferred with separation of model parameters for each of the two genes and for all three codon positions of gGAPDH gene. Bayesian posterior probabilities (5 million generations) and maximum likelihood bootstrap values (1,000 replicates) are shown at the nodes. Dots mark branches with maximal statistical support. Dashes (-) indicate bootstrap support below 50% or different topology. The tree was rooted with sequences of *Paratrypanosoma confusum*. Double-crossed branches are at 50% of their original lengths. The scale bar denotes the number of substitutions per site.

Bayesian and maximum likelihood phylogenetic trees reconstructed using concatenated 18S rRNA and gGAPDH sequences were generally congruent and consistent with previously published ones (Fig 3). *C*. *thermophila* formed a monophyletic group with *C*. *insperata* and *Leptomonas bifurcata*. This clade is distant from that including *C*. *fasciculata* (syn. *C*. *luciliae*), whose sister species is *C*. *dedva*.

### Taxonomic summary

Class **Kinetoplastea** (Honigberg, 1963)

Subclass **Metakinetoplastina** Vickerman, 2004

Order **Trypanosomatida** (Kent, 1880)

Family **Trypanosomatidae** (Doflein, 1901)

Genus ***Crithidia*** Léger, 1904

***Crithidia thermophila*** (Roitman et al., 1977) emend. Kostygov, d'Avila-Levy et Yurchenko, 2017.

**Synonyms**: *C*. *luciliae thermophila* Roitman et al., 1977; *C*. *confusa* Maslov et Lukeš, 2009; *C*. *deanei* Carvalho, 1973 (in part, see remark 2).

**Type host**: *Zelus leucogrammus* (Perty, 1833) (Hemiptera: Reduviidae).

**Type location**: Goiânia, Brazil.

**Neotype**: reference culture COLPROT 054 (= ATCC 30817).

**Diagnosis**: corresponds to that of *C*. *confusa* (see remark 3).

**Sequences**: EU079129, JF717837, KY264937 (18S rRNA), JF717832, KY264930—KY264936 (gGAPDH), JF734887, KY264921—KY264929 (SL RNA).

**Remarks**: 1) The name *Crithidia thermophila* is prioritized over the name *C*. *confusa* because of the chronology of species description and in accordance with the article 23.3.1 of the International Code of Zoological Nomenclature. 2) Isolate ATCC 30818 of *C*. *hutneri* and former aposymbiotic strain ATCC 30969 (*C*. *deanei*) derived from culture ATCC 30255 (*Angomonas deanei*) also belongs to this species. 3) Comprehensive taxonomic description was already made for *C*. *confusa* [20] now synonymized with *C*. *thermophila*. 4) Original description of *C*. *luciliae thermophila* by Roitman et al. cannot be used for proper species identification.

***Crithidia fasciculata*** Léger, 1904

**New synonym**: *C*. *luciliae* (Strickland, 1911) Wallace et Clark, 1959

### Growth kinetics and cell morphology at elevated temperature

**Fig 4:**
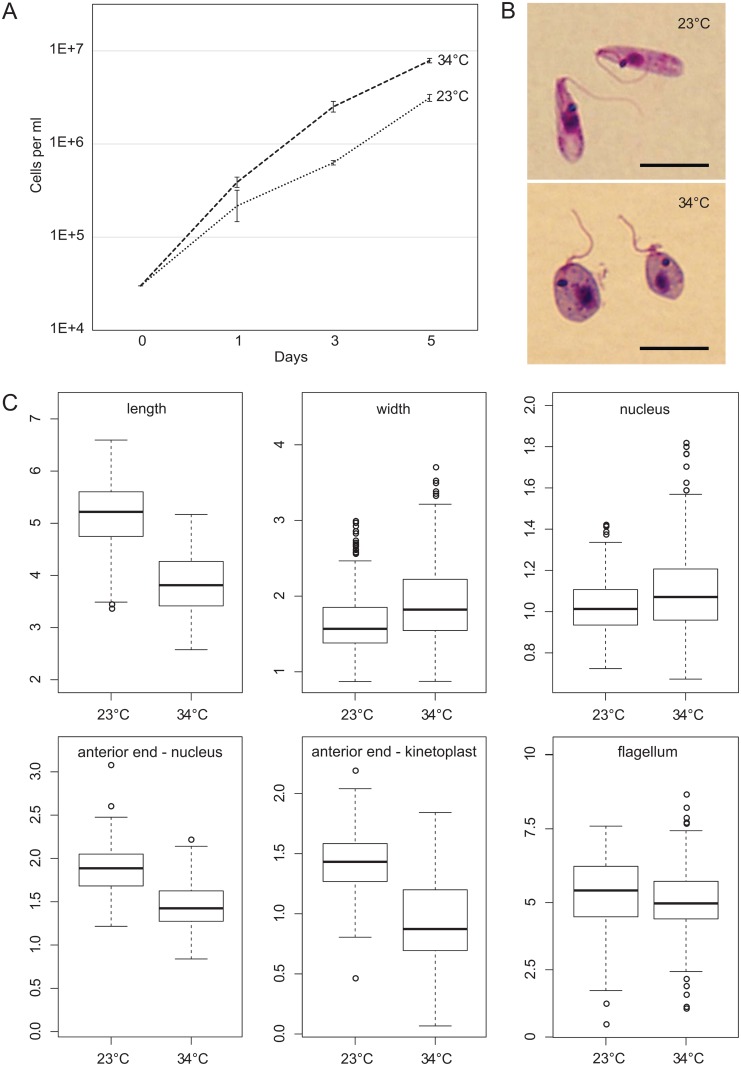
Comparison of growth of *Crithidia thermophila* (isolate COLPROT 054) at 23°C and 34°C. **(A)** Growth curves; **(B)** morphology on Giemsa-stained smears, scale bars are 5 μm; **(C)** morphometry of cells. Boxplots are from three independent biological replicates (50 cells per replicate) and show 1^st^ quartile, median, and 3^rd^ quartile, and 1.5 x interquartile range values. All measurements are in μm.

*C*. *thermophila* demonstrated a significant increase in division rate when cultivated at elevated temperature (Fig 4), confirming previous observations [35]. While the predominant morphotype at 23°C was a promastigote, at 34°C there were mainly choanomastigote-like cells (shorter, oval-shaped, with nucleus and kinetoplast relocated closer to the anterior end, Fig 4). In contrast to similar studies in *Leptomonas seymouri* [13], we did not observe changes in the flagellum length.

### Differential gene expression analysis

We identified 108 transcripts of *C*. *thermophila* differentially expressed at low and high temperature (S1 Table). Functional annotations were found for 71 of them. Genes up-regulated at elevated temperature (N = 86) belonged to 56 orthogroups (OGs), and 10 OGs were identified for down-regulated genes (N = 22). Some transcripts were not assigned to any OG, and most of them had no BLAST hits. Intriguingly, the number of genes differentially expressed at high temperature in *C*. *thermophila* is significantly smaller than in *L*. *seymouri* (see [13]). Only a few up-regulated genes were shared between the two species. These genes belonged to 6 OGs with the following annotations: i) putative fatty-acid desaturase, ii) putative β-fructofuranosidase, iii) NAD-dependent glycosomal glycerol-3-phosphate dehydrogenase, iv) paraflagellar rod protein, v) hypothetical protein [*L*. *pyrrhocoris*], and vi) hypothetical protein [*Leptomonas seymouri*].

Based on the available data, we were not able to predict functional roles of the hypothetical proteins. Therefore, we focused on the genes within the first four OGs and analyzed their putative roles in thermoresistance along with associated genes of the same pathways. Interestingly, not all genes involved in those pathways were documented as differentially expressed. We presume that it may be due to the differences in mechanisms regulating gene expression [57].

Fatty-acid desaturases are enzymes introducing double bonds into the fatty acyl chains. Overexpression of these enzymes seem to play a role in membrane-lipid reorganization which is a typical feature of response to a thermal stress [58,59].

Several enzymes involved in sugar metabolism were also upregulated. One of them is β-fructofuranosidase, which cleaves the disaccharide sucrose into glucose and fructose and makes hexose sugars available for oxidation by the glycolytic pathway. Increased expression of fructose-1,6-bisphosphate aldolase (producing triose phosphates dihydroxyacetone phosphate and glyceraldehyde 3-phosphate from the fructose-1,6-bisphosphate for glycolysis and gluconeogenesis) and glycerol-3-phosphate dehydrogenase (an enzyme responsible for redox conversion of dihydroxyacetone phosphate to glycerol 3-phosphate) also points to glycolysis. In *T*. *brucei*, NADH produced in this process is oxidized by the glycosomal/mitochondrial triose-phosphate shuttle and the alternative oxidase (TAO). However, *Crithidia* spp. lacks TAO [60,61], and glycolytic NADH seems to be oxidized by the mitochondrial respiratory complex I (MRC I). This scenario is supported by upregulation of two subunits (NADH dehydrogenase subunit 7 and NADH-ubiquinone oxidoreductase chain 1) of MRC I and also agrees with the increased expression of phosphate permease, while the mitochondrial phosphate transporter is downregulated. Summing it up, we conclude that in *C*. *thermophila* glycolysis is enhanced at elevated temperature. This confirms previous observation of increased consumption of carbohydrates by *C*. *thermophila* under these conditions (Table 1 in [38]).

The paraflagellar rod proteins (PFRs) are main components of the paraflagellar rod indispensable for flagellar function [62,63]. PFR2 is the main structural component of paraflagellar rod necessary for its correct assembly [64]. Since the flagellum of *C*. *thermophila* cells does not elongate at elevated temperature, we speculate that the increased expression of *Pfr2* may reflect higher rate of cell division. Similar pattern of overexpression at increased temperature has been observed for α- and β-tubulins, the main components of the cytoskeleton and apparently can be explained by the same mechanism.

Besides its role in motility, flagellum is important for signal transduction. For example, Ca^2+^ plays an important role in *T*. *cruzi* and *L*. *amazonensis* differentiation and interaction with host cells [65]. An elevated transcription of the flagellar calcium-binding protein in *C*. *thermophila* may indicate that stress response triggered by high temperature can lead to the remodeling of biochemical apparatus.

Mitochondrial Hsp70 (mtHsp70) is overexpressed in *C*. *thermophila* at 34°C. The mtHsp70 is an organellar counterpart of the heat shock protein 70 and takes part in several essential processes, such as folding of the newly synthesized, damaged and aggregated proteins, and degradation of denatured and unstable proteins [66]. It is also involved in the Fe-S cluster biogenesis [67] and protein import across the organellar membranes [68]. In trypanosomatids mtHsp70 may also be involved in mitochondrial tRNA import [69] and plays an important role in kDNA replication and maintenance, the latter function is likely being retained from prokaryotes [70]. Overexpression of heat shock proteins is one of the universal cellular responses to temperature stress.

Two elongation factors (eEF1α and eEF1Bγ2) were overexpressed in *C*. *thermophila* at elevated temperature. In addition to their role in protein synthesis, these factors were also implicated in other processes. For example, eEF1Bγ of *Leishmania major* and *Crithidia fasciculata* was shown to possess trypanothione S-transferase activity in response to oxidative and xenobiotic stresses [71,72]. In trypanosomatids, trypanothione is central for detoxification which (along with trypanothione reductase and tryparedoxin) carries the reducing equivalents from NADPH to several peroxidases [73,74]. Thus, the increase of eEF1Bγ expression may correlate with elevated levels of the reactive oxygen species (ROS) in *C*. *thermophila* at high temperature. Surprisingly, and in contrast to *L*. *seymouri*, we did not observe changes in the expression of ROS-protecting enzymes. In particular, there was no increase in the RNA level of catalase, which presumably facilitated adaptation of Leishmaniinae to different environmental conditions after its acquisition from bacteria by horizontal gene transfer [75]. This implies that mechanisms of ROS protection may vary in different species of this group.

## Conclusions

The ability to survive and multiply at elevated temperature is a hallmark of dixenous *Leishmania* and *Trypanosoma*. In monoxenous trypanosomatids this feature is not very common. The only well-studied example in this respect has been *L*. *seymouri*, whose biology remains largely unknown. For comparison, here we analyzed the phenomenon of thermoresistance in a related species, *C*. *thermophila*. Differential gene expression analysis shows that it utilizes common stress-induced mechanisms in response to elevated temperature. At the same time, we documented accelerated metabolism correlating with increased rate of cell division. This implies that, justifying its species name, elevated temperature is optimal for *C*. *thermophila*, in contrast to *L*. *seymouri*, which experiences a real stress under these conditions. Therefore, dramatic differences in transcription profiles of the two species with only a few genes showing the same expression pattern likely reflect different mechanisms of thermotolerance. We also documented numerous genes with unknown function annotated as hypothetical proteins. We believe that studying these genes may shed light on the adaptations of trypanosomatids to elevated temperature.

## Supporting information

S1 Table Genes up- or down-regulated at elevated temperature in *Crithidia thermophila*. Annotation and orthologs IDs are provided when available.
(XLSX)
